# Growth Inhibition of Phaeocystis Globosa Induced by Luteolin-7-O-glucuronide from Seagrass Enhalus acoroides

**DOI:** 10.3390/ijerph16142615

**Published:** 2019-07-23

**Authors:** Jingyi Zhu, Han Xiao, Qi Chen, Min Zhao, Dong Sun, Shunshan Duan

**Affiliations:** Department of Ecology, College of Life Science and Technology, Jinan University, Guangzhou 510632, China

**Keywords:** harmful algal blooms, *Enhalus acoroides*, *Phaeocystis globosa*, Luteolin-7-O-glucuronide

## Abstract

*Enhalus acoroides* (*E. acoroides*) is one of the most common species in seagrass meadows. Based on the application of allelochemicals from aquatic plants to inhibit harmful algal blooms (HABs), we used *E. acoroides* aqueous extract against harmful algae species *Phaeocystis globosa* (*P. globosa*). The results showed that *E. acoroides* aqueous extract could significantly inhibited the growth of *P. globosa*, decrease the chlorophyll-a content and photosynthetic efficiency (Fv/Fm) values of *P. globosa*, followed by vacuolization, plasmolysis, and the destruction of organelles. Twelve types of major chemical constituents were identified in *E. acoroides* aqueous extracts by ultraperformance liquid chromatography-high resolution mass spectrometry (UPLC-HRMS), including six flavonoids, two homocyclic peptides, two long-chain aliphatic amides, one tannin, and one nitrogen heterocyclic compound. Flavonoids were the characteristic chemical constituents of *E. acoroides* aqueous extract. Furthermore, the antialgal activity of luteolin-7-O-glucuronide (68.125 μg/mL in 8 g/L *E. acoroides* aqueous extract) was assessed. The EC_50–96 h_ value was 34.29 μg/mL. In conclusion, the results revealed that luteolin 7-O-glucuronide was one of the antialgal compounds of *E. acoroides* aqueous extract, with potential application as novel algaecide.

## 1. Introduction

Harmful algal blooms (HABs) cause a series of ecological problems. They contribute to the death of fish, birds, and mammals (including humans), cause respiratory or digestive tract problems, as well as losses of coastal resources [1]. *Phaeocystis globosa* (*P. globosa*) is regarded as an unwanted algal species. It is associated with shellfish mortality and acid rain, and can produce hemolytic toxins, bad odors, and form foam through high biomass accumulation [2,3]. *P. globosa* blooms not only destroy marine ecosystem but also are hazardous to human health and aquaculture. One of the most striking characteristics of *P. globosa* is that they have a heteromorphic life cycle, with solitary cells as well as colonies. *P. globosa* blooms are frequently found in China, Viet Nam, and the eastern English Channel [4,5,6]. This inspired scientists to develop effective methods to inhibit and prevent the *P. globosa* blooms.

Biological methods, which rely on allelochemicals from aquatic plants to constrain harmful algal blooms, have become a research hotspot for their high biodegradability, low toxicity, and low cost [7,8,9]. Seagrasses are submerged marine aquatic plants that play an important role in coastal ecosystems such as providing food and habitat for animals, taking up metals, and contributing to the primary production [10]. Seagrasses are abundant in secondary metabolites, which is thought to be a defense mechanism that provides the plant with the capability to survive and resist different stress types, such as larvae feeding, fungal and bacterial infection [11]. Many studies reported that secondary metabolites from seagrasses have antibacterial, antiviral, anti-fungal, anti-inflammatory, and antioxidant activities, but only a few studies have examined the antialgal activity of seagrasses for biological application [12,13]. Consequently, there is a need to explore the antialgal activity of seagrasses.

*Enhalus acoroides* (*E. acoroides*) is a morphologically large, long-lived, and common seagrass [14]. In addition to being edible, *E. acoroides* has been used as a remedy against stings, and is also effective for muscle pains, wounds, and stomach problems [13]. Interestingly, red tides rarely occur in places where *E. acoroides* grows, and the seawater is very clear in such places [15]. Previous studies reported that *E. acoroides* synthesized flavonoids to resist larvae feeding and bacterial infection, which implied that the seagrass may have inhibitory effects on algae [16]. Hence, further studies are needed to examine whether *E. acoroides* produces secondary metabolites with antialgal activity leading to the rarity of red tides in places where *E. acoroides* grows. In order to develop antialgal compounds from *E. acoroides*, we investigated the inhibitory effects of *E. acoroides* aqueous extracts on the growth of *P. globosa*, identified the chemical constituents, and uncovered the antialgal compounds.

## 2. Materials and Methods

### 2.1. Materials and Culture Conditions

*E. acoroides* were collected from Xincun bay, ling shui county, Hainan province (18°23′ N, 109°59′ E), and stored in a heat insulated box at room temperature (about 4 °C). *P. globosa* were obtained from the Research Center of Hydrobiology, Jinan University, Guangzhou, China. Algal cells were incubated in F/2 medium under a photoperiod of 12 h (light):12 h (dark) at 22–24 °C and an irradiance of 100 μmol photons m^−2^s^−1^.

### 2.2. Preparation of E. acoroides Aqueous Extract

The seagrass was cleaned with distilled water and scrubbed with a soft brush to remove algae and other microorganisms attached to its surface as much as possible. Then, the cleaned seagrass was dried in a drying oven at 65 °C to constant weight. After drying, the seagrass was ground into powder, filtered by a 40-mesh sieve, mixed with seawater (1:50 w/v), and placed in a constant temperature water bath electromagnetic stirring pot of 25 °C for 48 h at middle-speed. The aqueous extract was then centrifuged for 15 min at 5000 rpm. The supernatant was filtered with 0.22 μm filter to remove all microorganisms and was stored at 4 °C before use.

### 2.3. Treatments with E. acoroides Aqueous Extract

To study the inhibitory effects of different concentrations of *E. acoroides* aqueous extract on the growth of *P. globosa*, conical flasks (50 mL) were prepared and autoclaved. Each group of flasks contained 20 mL culture media. The aqueous extract concentration in the culture medium in each group was 0, 2, 4, 6, and 8 g/L. The group without aqueous extract served as the control sample. The initial algal density (IAD) in each flask was 1 × 10^5^ cells/mL, and each flask was inoculated with *P. globosa* culture in the exponential growth phase. Each treatment was replicated three times. The cultures were incubated under the conditions mentioned above. To obtain the algal growth curve under treatments with different concentrations of aqueous extract, the algal cells were counted from 0 to 96 h under a light microscope with a hemocytometer.

The inhibition rate (IR) of *P. globosa* growth under treatment of different concentrations of *E. acoroides* aqueous extract was calculated by the following equation:

IR = (N_0_ − N))/N_0_ × 100%, (1)

where N is the Algal density of the treatment group (cell/mL); N_0_ is the Algal density of the control group (cell/mL).

### 2.4. Chlorophyll-a Concentration and Photosynthetic Efficiency (Fv/Fm) Assays

Chlorophyll-a is the primary photosynthetic pigment. Chlorophyll-a concentration was measured according to previous methods [17]. Briefly, the algal cells were collected and centrifuged. The pigments were extracted using 95% ethanol at 4 °C in the dark for 48 h. After extraction, the samples were centrifuged for 30 min at 2500× *g*. The supernatant was used to measure absorbance values at 665 nm and 645 nm. The pigments were calculated according to the following formula:

Chlorophyll-a (mg/L) = 12.7 × A_665_ − 2.69 × A_645_, (2)

The chlorophyll fluorescence parameter, Fv/Fm is used to reflect the maximum quantum efficiency of PSII photochemistry. A Plant Efficiency Analyzer (PEA MK2, Hansatech Instrument LTD, Norfolk, UK) was used to measure Fv/Fm. The algal cultures (2 mL) were incubated in the dark for 20 min and then Fv/Fm was measured. 

### 2.5. Transmission Electron Microscopy (TEM) Analysis

The algal cells were collected (2000× *g*, 15 min) and then fixed overnight at 4 °C in PBS buffer containing 2.5% glutaraldehyde (v/v). The fixed algal cells were washed three times in PBS buffer (50 mM, pH 7.4) and post-fixed for 2 h in 1% OsO_4_ in the same buffer at room temperature. After being washed three times, the samples were dehydrated through a graded ethanol series (30, 50, 70, 80, 85, 90, and 100% (v/v in ddH_2_O); 15–20 min at each concentration), then permeated and embedded in araldite resin. Sections (80–100 nm), obtained with an ultramicrotome, were stained in 3% acetic acid uranium-citric acid and viewed using TEM.

### 2.6. Ultraperformance Liquid Chromatography-High Resolution Mass Spectrometry (UPLC-HRMS) Analysis

UPLC-HRMS analysis were performed with an Acquity UPLC I-class system (Waters Corporation, Milford, MA, USA), and an Acquity BEH C18 (Waters Corporation, Milford, MA, USA) column (1.7 μm, 100 mm × 2.1 mm) was used for separation. The mobile phase was composed of acetonitrile (A) and water (0.1% formic acid) (B). The column oven was set at 40 °C. The flow rate was 0.3 mL/min and the injection volume was set at 1.0 μL. The gradient used was as follows: 0.00–1.00 min, 10.0% (A); 1.00–3.00 min, 10.0–35.0% (A); 3.00–6.00 min, 35.0–65.0% (A); 6.00–9.00 min, 65.0–85.0% (A); 9.00–10.00 min, 85.0–90.0% (A); 10.00–11.00 min, 90.0–10.0% (A); 11.00–13.00 min, 10.0% (A). An electrospray ionization (ESI) system and A Xevo G2-S Q Tof time-of-flight mass spectrometer (Waters Corporation, Milford, MA, USA) were used. Optimal HRMS parameters were as follows: the capillary voltage was 2.5 kV, the ion source temperature was 120 °C, the cone voltage was 25 V, the desolvation gas temperature was set at 400 °C and the desolvation gas flow was set at 1000 L/h. The data was processed by Masslynx 4.1 software and the structure of the chemical compositions was analyzed by the UNIFI^®^ Scientific Information System (Waters Corporation, Milford, MA, USA).

### 2.7. Treatments with Luteolin-7-O-glucuronide and Quantification of Luteolin-7-O-glucuronide

Luteolin-7-O-glucuronide (96% purity) was purchased from Chengdu Push Bio-Technology Co., Ltd (Chengdu, China). The solution of luteolin-7-O-glucuronide was filtered with 0.22 μm filter before use. Antialgal activity of luteolin-7-O-glucuronide was measured at concentrations of 0, 20, 40, 60, 80, and 100 µg/mL. Each treatment was triplicated. The IAD was 1 × 10^5^ cells/mL. The culture conditions were as described above. The algal cells were counted from 0 to 96 h under a light microscope with a hemocytometer. The IR was calculated by the above mentioned equation.

The quantification of luteolin-7-O-glucuronide was performed using external calibration. Compound was dissolved in methanol using a vortex mixer to prepare standard solutions (1 µg/mL) and the 8 g/L *E. acoroides* aqueous extract was diluted to accurately measure the compound. The injection volume was 1.0 μL. UPLC-HRMS conditions were as described above. The results are presented as µg per mL.

### 2.8. Data Analysis

Statistical analysis of data was performed using GraphPad Prism 7 software. The means and standard deviations (SD) of all data were determined and graphed. Students’ *t*-test was used, and *p* < 0.05 and *p* < 0.01 were considered significant. 

## 3. Results

### 3.1. Effects of E. acoroides Aqueous Extract on the Growth of P. globosa

**Figure 1:**
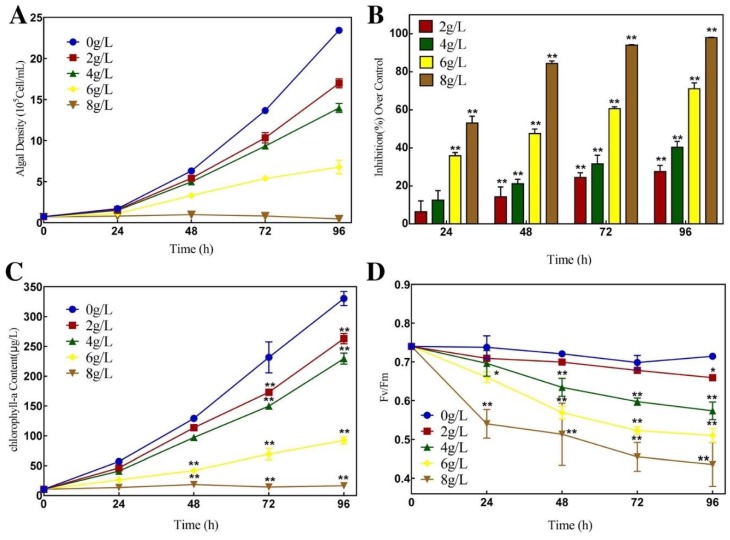
Effects of *E. acoroides* aqueous extract on (**A**) growth curve; (**B**) inhibition rate; (**C**) chlorophyll-a concentration; (**D**) photosynthetic efficiency (Fv/Fm). Data are means ± SD (n = 3). * *p* < 0.05, ** *p* < 0.01 indicate significant differences.

The relationship between the concentration of aqueous extract and the growth of *P. globosa* is shown in Figure 1A. After 48 h, the inhibitory effects of *E. acoroides* aqueous extract concentrations from 2 g/L to 8 g/L on *P. globosa* were significant (*p* < 0.01). Also, the IR increased with the increase in aqueous extract concentration and exposure time (Figure 1B). At 96 h, the IR of *P. globosa* exposed to 2, 4, 6, and 8 g/L of *E. acoroides* aqueous extract peaked, which were 27.52% (*p* < 0.01), 40.32% (*p* < 0.01), 71.10% (*p* < 0.01), and 98.02% (*p* < 0.01), respectively.

### 3.2. Effects of E. acoroides Aqueous Extract on Chlorophyll-a Concentration and Fv/Fm of P. globosa

As shown in Figure 1C, there was significant difference (*p* < 0.01) between the treatment groups of 2, 4, 6, and 8 g/L and the controls after 72 h. The chlorophyll-a content reached the highest in the experimental groups of 2, 4, and 6 g/L after 96 h, and were 79.60% (*p* < 0.01), 69.47% (*p* < 0.01), and 27.91% (*p* < 0.01) compared to the controls, respectively. The chlorophyll-a content in the group of 8 g/L remained at a low level, and was 4.96% (*p* < 0.01) as compared to the control after 96 h. The Fv/Fm values in the treatment groups of 2, 4, 6, and 8 g/L showed a downward trend with prolonged exposure time (Figure 1D). At 96 h, the values reached the lowest, and were 96.92% (*p* < 0.01), 80.32% (*p* < 0.01), 71.41% (*p* < 0.01), and 60.95% (*p* < 0.01) compared to the controls after exposure to the *E. acoroides* aqueous extract at concentrations of 2, 4, 6, and 8 g/L, respectively.

### 3.3. Effects of E. acoroides Aqueous Extract on Subcellular Structure of P. globosa

**Figure 2:**
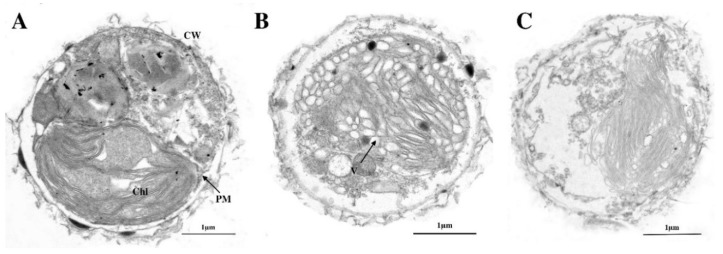
Ultrastructure of *P. globosa* after treatment with 8 g/L of *E. acoroides* aqueous extract. (**A**) Control, (**B**) 48 h, (**C**) 96 h. Chl, chloroplast; PM, plasma membrane; V, vacuole; CW, cell wall.

Transmission electron microscopy (TEM) revealed modifications in the ultrastructure of *P. globosa* due to the effects of *E. acoroides* aqueous extract. Figure 2A shows that the algal cells in the control had intact cellular morphology and microscopic structure. Figure 2B shows obvious plasmolysis and vacuolization. Many organelles were severely damaged at this time point. Considerable changes appeared in the chloroplasts, especially in the loose lamellar structures, which was not as compact as before. After 96 h exposure, the plasma membrane was severely damaged and cellular inclusions were released from cells, indicating the death of algal cells (Figure 2C). 

### 3.4. Chemical Constituent Analysis of Aqueous Extract from E. acoroides

**Figure 3:**
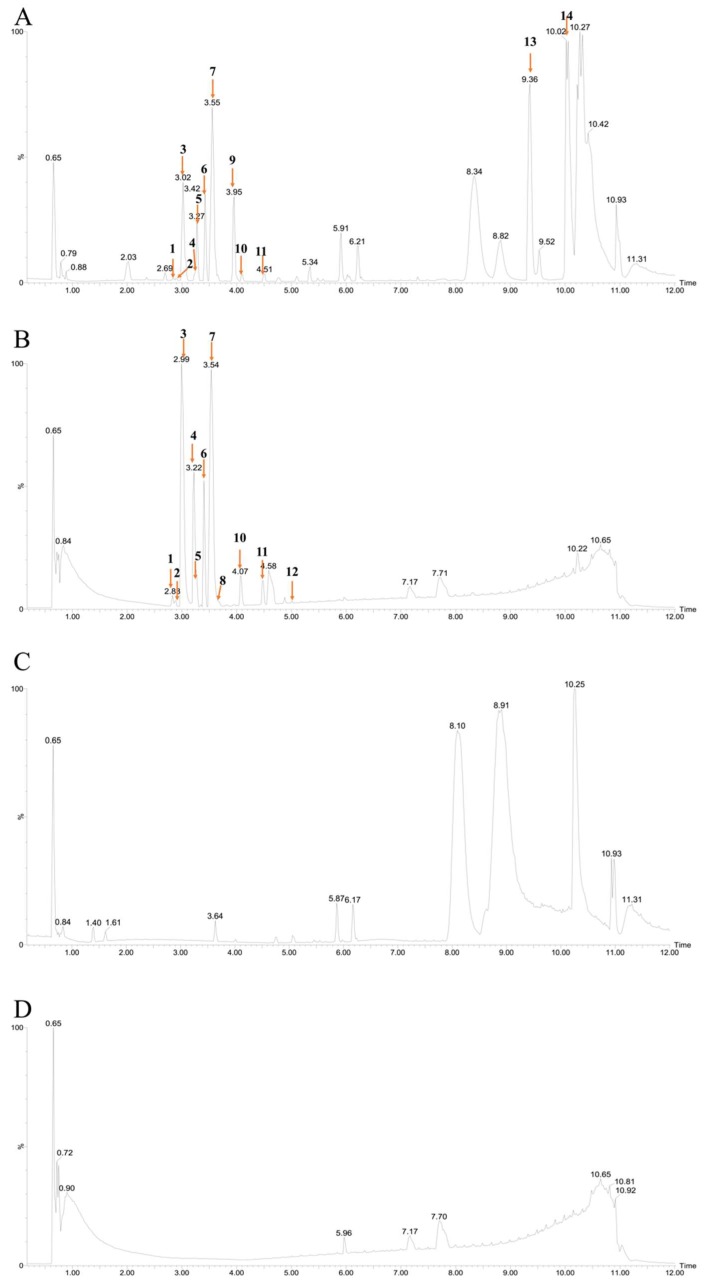
Identification of chemical constituents from *E. acoroides* aqueous extracts in (**A**) positive ionization mode; (**B**) negative ionization mode; identification of chemical constituents from sterilized artificial seawater in (**C**) positive ionization mode; (**D**) negative ionization mode. Numbers above peaks represent retention times, in minutes.

**Figure 4:**
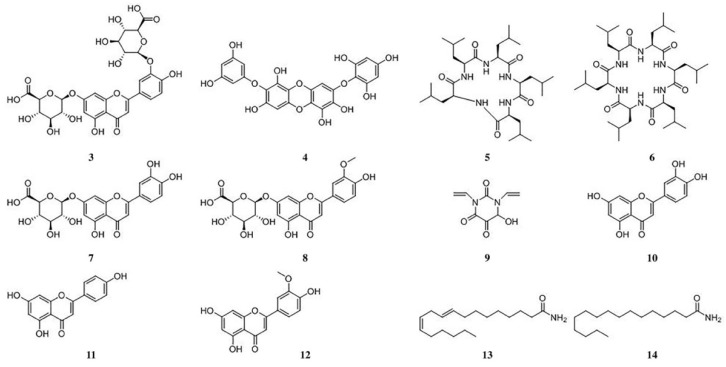
Chemical structures of compounds **3**–**14** (compounds **1** and **2** are unknown).

In order to identify the compounds in *E. acoroides* aqueous extract, positive and negative ionization modes were used. Table 1 and Figure 3 indicated that fourteen peaks were detected from *E. acoroides* aqueous extract, excluding the compounds in the sterilized artificial seawater (Figure 3C,D). Briefly, a total of 12 major chemical constituents were identified (Figure 4), and mass spectrograms of all identified compounds are presented in Figures S2–S15. Compounds 1 and 2 were identified as polypeptides, but the specific structures could not be accurately inferred due to their low contents in *E. acoroides* aqueous extract and the poor information of secondary ion mass spectrometry. Among the 12 identified compounds, there were six flavonoids, two homocyclic peptides, two long-chain aliphatic amides, one tannin, and one nitrogen heterocyclic compound.

### 3.5. Antialgal Activity of Luteolin-7-O-glucuronide

Six of twelve major chemical constituents were flavonoids, indicating that flavonoids were the characteristic chemical constituents of *E. acoroides* aqueous extract. Many studies have demonstrated that flavonoids have great antialgal activity [18]. Therefore, we were mainly focused on flavonoids in our study. Among the flavonoids, the peak area of luteolin-3′,7-O-diglucuronide was the largest (430,617) in Table 2, followed by luteolin-7-O-glucuronide (268,581). There was no direct relation between concentrations and peak area, because the ionization difficulty and ionic stability of different compounds were different. However, we could compare concentrations of flavonoid compounds in aqueous extract based on peak area, because similar classes of compounds may possess similar chemical features and ionization potential [19]. Therefore, we speculated that luteolin-3′,7-O-diglucuronide and luteolin-7-O-glucuronide may have relatively higher concentrations compared to other flavonoids in aqueous extract, which might have inhibitory effects on *P. globosa*.

Luteolin-7-O-glucuronide has good prospects for application compared to luteolin-3′,7-O-diglucuronide. Luteolin-7-O-glucuronide can treat diseases related to reactive species production and oxidative stress and can resist to bacterial infections, thus it may have inhibitory effects on *P. globosa* [20,21]. Yet, there are no studies on the activity of luteolin-3′,7-O-diglucuronide. Therefore, luteolin-7-O-glucuronide was selected as the novel algaecide to control *P. globosa* blooms and the inhibitory effects of luteolin-7-O-glucuronide on *P. globosa* were investigated in our research.

**Figure 5:**
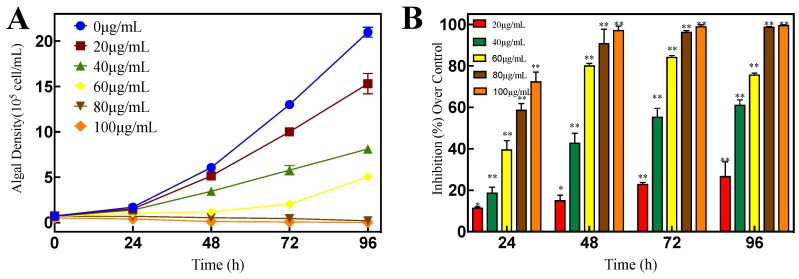
Effects of luteolin-7-O-glucuronide on the algal density of *P. globosa* (**A**) growth curve; (**B**) inhibition rate. Data are means ± SD (n = 3). * *p* < 0.05, ** *p* < 0.01 indicate significant differences.

Figure 5A shows that the algal density of *P. globosa* was significantly decreased (*p* < 0.01) after 48 h exposure to different concentrations of luteolin-7-O-glucuronide. The IR increased with the increase of luteolin-7-O-glucuronide concentration and exposure time (Figure 5B). After 96 h, the IR was 26.92% (*p* < 0.01), 61.28% (*p* < 0.01), 75.88% (*p* < 0.01), 99.02% (*p* < 0.01), and 99.78% (*p* < 0.01) after exposure to luteolin-7-O-glucuronide at concentrations of 20, 40, 60, 80, and 100 μg/mL, respectively.

## 4. Discussion

Many studies have used the plant aqueous extract, organic extract, thalli, culture filtrate, fresh tissue, dry tissue, and dry powder to control HABs [22,23,24,25,26]. However, only a few studies have uncovered antialgal compounds from plants [27,28,29]. In the present study, *E. acoroides* aqueous extract had a notable inhibitory effect on *P. globosa*, which was concentration-dependent. Therefore, the antialgal activities of *E. acoroides* aqueous extract were believed to indicate the existence of antialgal active substances. To explore any damage to the photosynthetic system and subcellular structure of *P. globosa*, further experiments were conducted. Chlorophyll-a content can be used to reflect potential photosynthesis capacity and estimate the biomass of algae, and Fv/Fm was used to evaluate photosynthesis efficiency [30]. Based on the decrease in chlorophyll-a concentration and Fv/Fm values, we concluded that the growth and reproduction of *P. globosa* was inhibited and its photosynthetic system was destroyed, which was also confirmed by the seriously damaged chloroplast. Under TEM, the lamellar structures of chloroplasts became looser with exposure time, finally breaking up. Other obvious differences could be observed between the treated and untreated cells, such as vacuolization, organelle decomposition, and plasmolysis, finally leading to the death of treated cells. Previous studies have reported that prodigiosin and the supernatant of bacterium inhibited the growth of *P. globosa* and destructed photosynthetic system and subcellular structure, which were consistent with our findings [17,31,32].

Our research indicated that *E. acoroides* aqueous extract could be used to control HABs. Nevertheless, considering that *E. acoroides* grows along the coastline, between 40 and 160m from the shore, these plants are not easily assessed [33]. Thus, the following step is to investigate whether the antialgal compounds from *E. acoroides* could be found in other plants that are easier to access. In this study, we used UPLC-HRMS to identify the chemical constitutes in *E. acoroides* aqueous extract, including six flavonoids, two homocyclic peptides, two long-chain aliphatic amides, one tannin, and one nitrogen heterocyclic compound. There were many types of flavonoids in *E. acoroides* aqueous extract compared to other types of compounds, which was similar to a previous report [16]. Our research revealed that flavonoids were the characteristic chemical constituents of *E. acoroides* aqueous extract and according to the literature flavonoids usually have good antialgal activity [18]. Thus, in this study, luteolin-7-O-glucuronide was selected as the representative flavonoids to investigate the potential antialgal activity. The EC_50–96h_ of luteolin-7-O-glucuronide was 34.29 μg/mL, indicating that it was an effective algaecide. The concentration of luteolin-7-O-glucuronide was 68.125 μg/mL in 8 g/L *E. acoroides* aqueous extract (Table 3), which means that approximately 80.03% *P. globosa* was constrained at this concentration at 96 h (Figure S1). Combined with the IR of 8 g/L *E. acoroides* aqueous extract, which was 99.78% at 96 h (Figure 1B), we concluded that luteolin-7-O-glucuronide played a role in the inhibitory effects of *E. acoroides* aqueous extract on the growth of *P. globosa*. However, it was difficult to attribute the antialgal activity to a specific compound in such a complex aqueous extract, because of the existence of synergistic and antagonistic effects [34].

This is the first study that reported on the antialgal activity of luteolin-7-O-glucuronide, which can be used as an algicide to control *P. globosa* blooms. It is widely present in lots of plants and has significant inhibitory effects on *P. globosa* [35,36]. Zhang reported that 5 µg/mL prodigiosin, from bacterium *Hahella*, produced 84% algicidal activity in 72 h [37]. Although the inhibitory effects of prodigiosin are superior, it is insoluble in water and expensive. It is also difficult to be used to control *P. globosa* blooms in natural water bodies. The application of secondary metabolites, from algicidal bacteria, against *P. globosa* has been reported [32]. However, it remains unclear whether secondary metabolites can have negative effects on higher trophic levels. In general, antialgal activity compounds from aquatic plants may have little effect on other organisms and aquatic ecosystems. Previous reports have shown that luteolin-7-O-glucuronide had antioxidant, anti-mutagenic, anti-genotoxic, anti-inflammatory, and anti-arthritic activities, which are beneficial for humans [38,39,40]. Therefore, the application of luteolin-7-O-glucuronide may be safer.

## 5. Conclusions

In summary, this study revealed that *E. acoroides* aqueous extract had significant inhibitory effects on *P. globosa*. *E. acoroides* aqueous extract inhibited algal growth by decreasing its chlorophyll-a content, Fv/Fm values, and by damaging its subcellular structure. UPLC-HRMS analysis revealed that flavonoids were the characteristic chemical constituents. Among the flavonoids, luteolin-7-O-glucuronide was found to have significant inhibitory effects on the growth of *P. globosa*. The antialgal compound can be used as a novel algicide to control *P. globosa* blooms, which is widely present in lots of plants and non-toxic for other organisms.

However, our research is limited, thus additional studies are required. The cost of adding luteolin-7-O-glucuronide for a typical treatment for *P. globosa* blooms is still unknown. The antialgal mechanism need to be further elucidated in order to reveal the practical utilization values.

## Figures and Tables

**Table 1:** Identification of chemical constituents from *E. acoroides* aqueous extracts.

No.	Rt (min)	Exact Mass (m/z) [M + H]^+^ [M − H]^−^		Types	Molecular Formula	Name of the Chemical Constituent
1	2.83	614.1519	612.1376	polypeptides		polypeptide (unknown)
2	2.90	614.1519	612.1376	polypeptides		polypeptide (unknown)
3	3.00	639.1227	637.1060	flavonoids	C_27_H_26_O_18_	luteolin-3′,7-O-diglucuronides
4	3.22	513.0708	511.0551	tannins	C_24_H_16_O_13_	diphlorethohydroxycarmalol
5	3.27	566.4286	610.4195	homocyclic peptides	C_30_H_55_N_5_O_5_	cyclo (l-leucyl-l-leucyl-l-leucyl-l-leucyl-l-leucyl)
6	3.42	679.5132	723.5030	homocyclic peptides	C_36_H_66_N_6_O_6_	cyclo (l-leucyl-l-leucyl-l-leucyl-l-leucyl-l-leucyl-l-leucyl)
7	3.55	463.0871	461.0717	flavonoids	C_21_H_18_O_12_	luteolin-7-O-glucuronide
8	3.66		475.0868	flavonoids	C_22_H_20_O_12_	chrysoeriol-7-O-glucuronide
9	3.95	183.0794		nitrogen heterocyclic compounds	C_8_H_8_N_2_O_4_	6-hydroxy-1,3-divinyldihydropyrimidine-2,4,5(3*H*)-trione
10	4.07	287.0548	285.0419	flavonoids	C_15_H_10_O_6_	luteolin
11	4.48	271.0620	269.0449	flavonoids	C_15_H_10_O_5_	apigenin
12	5.18		299.0556	flavonoids	C_16_H_12_O_6_	chrysoeriol
13	9.36	280.2655		aliphatic amides	C_18_H_33_NO	(9*E*,12*Z*)-octadeca-9,12-dienamide
14	10.04	256.2672		aliphatic amides	C_16_H_33_NO	palmitamide

**Table 2:** Summary of the peak area of flavonoids from *E. acoroides* aqueous extracts.

No.	Rt (min)	Types	Molecular Formula	Name of the Chemical Constituent	Peak Area
3	3.00	flavonoids	C_27_H_26_O_18_	luteolin-3′,7-O-diglucuronides	430,617
7	3.55		C_21_H_18_O_12_	luteolin-7-O-glucuronide	268,581
8	3.66		C_22_H_20_O_12_	chrysoeriol-7-O-glucuronide	623
10	4.07		C_15_H_10_O_6_	luteolin	48,305
11	4.48		C_15_H_10_O_5_	apigenin	41,554
12	5.18		C_16_H_12_O_6_	chrysoeriol	4996

**Table 3:** Quantification of luteolin-7-O-glucuronide in 8g/L *E. acoroides* aqueous extract.

Rt (min)	Molecular Formula	Compound	Content (μg/mL)
3.55	C_21_H_18_O_12_	luteolin-7-O-glucuronide	68.125

## Supplementary Materials

The following are available online at https://www.mdpi.com/1660-4601/16/14/2615/s1, Figure S1: Correlation relationships between the contents of luteolin-7-O-glucuronide and the inhibition rate at 96 h, Figure S2: Mass spectrogram of polypeptide (unknown) at 2.83 min (A) positive ionization mode at low energy channels; (B) negative ionization mode at low energy channels; (C) positive ionization mode at high energy channels; (D) negative ionization mode at high energy channels, Figure S3: Mass spectrogram of polypeptide (unknown) at 2.90 min (A) positive ionization mode at low energy channels; (B) negative ionization mode at low energy channels; (C) positive ionization mode at high energy channels; (D) negative ionization mode at high energy channels, Figure S4: Mass spectrogram of luteolin-3‘,7-O-diglucuronides at 3.00 min (A) positive ionization mode at low energy channels; (B) negative ionization mode at low energy channels; (C) positive ionization mode at high energy channels; (D) negative ionization mode at high energy channels, Figure S5: Mass spectrogram of diphlorethohydroxycarmalol at 3.22 min (A) positive ionization mode at low energy channels; (B) negative ionization mode at low energy channels; (C) positive ionization mode at high energy channels; (D) negative ionization mode at high energy channels, Figure S6: Mass spectrogram of cyclo (L-leucyl-L-leucyl-L-leucyl-L-leucyl-L-leucyl) at 3.27 min (A) positive ionization mode at low energy channels; (B) negative ionization mode at low energy channels; (C) positive ionization mode at high energy channels; (D) negative ionization mode at high energy channels, Figure S7: Mass spectrogram of cyclo (L-leucyl-L-leucyl-L-leucyl-L-leucyl-L-leucyl-L-leucyl) at 3.42 min (A) positive ionization mode at low energy channels; (B) negative ionization mode at low energy channels; (C) positive ionization mode at high energy channels; (D) negative ionization mode at high energy channels, Figure S8: Mass spectrogram of luteolin-7-O-glucuronide at 3.55 min (A) positive ionization mode at low energy channels; (B) negative ionization mode at low energy channels; (C) positive ionization mode at high energy channels; (D) negative ionization mode at high energy channels, Figure S9: Mass spectrogram of chrysoeriol-7-O-glucuronide at 3.66 min negative ionization mode at low energy channels, Figure S10: Mass spectrogram of 6-hydroxy-1,3-divinyldihydropyrimidine-2,4,5(3H)-trione at 3.95 min (A) positive ionization mode at low energy channels; (B) postive ionization mode at high energy channels, Figure S11: Mass spectrogram of luteolin at 4.07 min (A) positive ionization mode at low energy channels; (B) negative ionization mode at low energy channels; (C) positive ionization mode at high energy channels; (D) negative ionization mode at high energy channels, Figure S12: Mass spectrogram of apigenin at 4.48 min (A) positive ionization mode at low energy channels; (B) negative ionization mode at low energy channels; (C) positive ionization mode at high energy channels; (D) negative ionization mode at high energy channels, Figure S13: Mass spectrogram of chrysoeriol at 5.18 min (A) negative ionization mode at low energy channels; (B) negative ionization mode at high energy channels, Figure S14: Mass spectrogram of (9E,12Z)-octadeca-9,12-dienamide at 9.36 min (A) positive ionization mode at low energy channels; (B) positive ionization mode at low energy channels, Figure S15: Mass spectrogram of palmitamide at 10.04 min (A) positive ionization mode at low energy channels; (B) positive ionization mode at low energy channels.
