# Supplementary material for: Growth Inhibition of Phaeocystis Globosa Induced by Luteolin-7-O-glucuronide from Seagrass Enhalus acoroides
Source: Int J Environ Res Public Health. 2019 Jul 23;16(14):2615. doi: 10.3390/ijerph16142615 (PMC6678453; doi:10.3390/ijerph16142615)
Supplement: Supplementary file 1 [file ijerph-16-02615-s001.pdf]

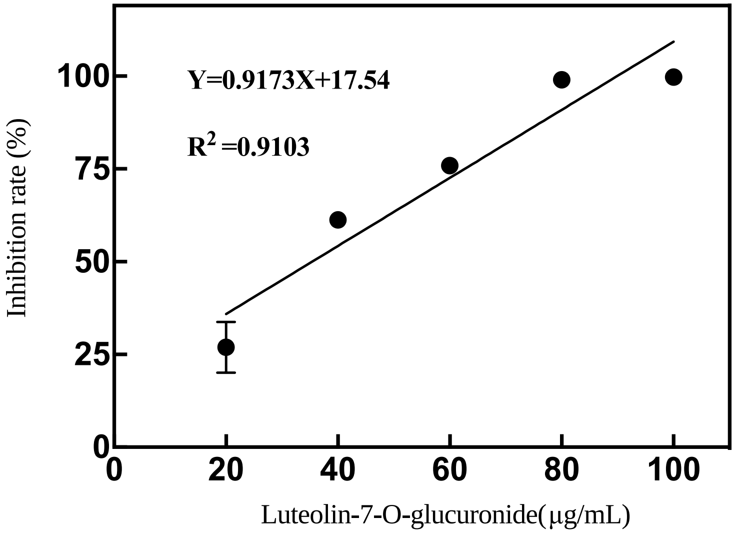

Figure S1. Correlation relationships between the contents of luteolin-7-O-glucuronide and the inhibition rate at 96 h.

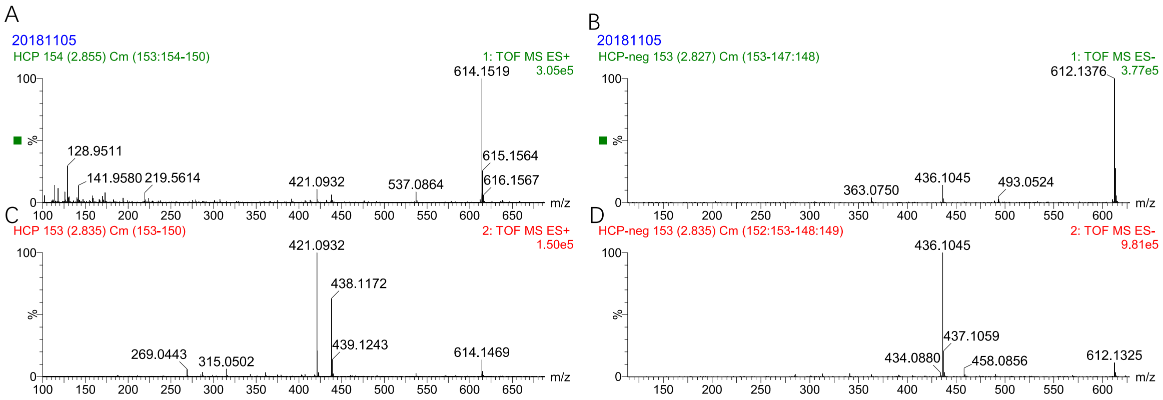

Figure S2. Mass spectrogram of polypeptide (unknown) at 2.83 min (**A**) positive ionization mode at low energy channels; (**B**) negative ionization mode at low energy channels; (**C**) positive ionization mode at high energy channels; (**D**) negative ionization mode at high energy channels.

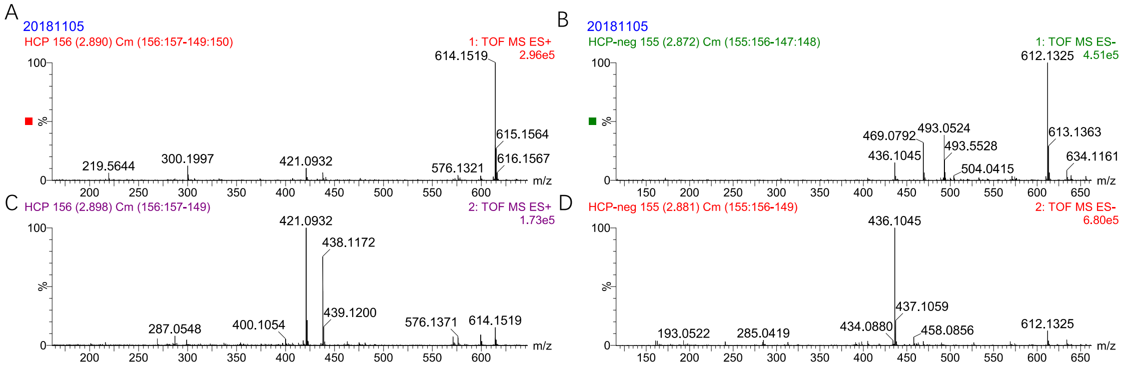

Figure S3. Mass spectrogram of polypeptide (unknown) at 2.90 min (**A**) positive ionization mode at low energy channels; (**B**) negative ionization mode at low energy channels; (**C**) positive ionization mode at high energy channels; (**D**) negative ionization mode at high energy channels.

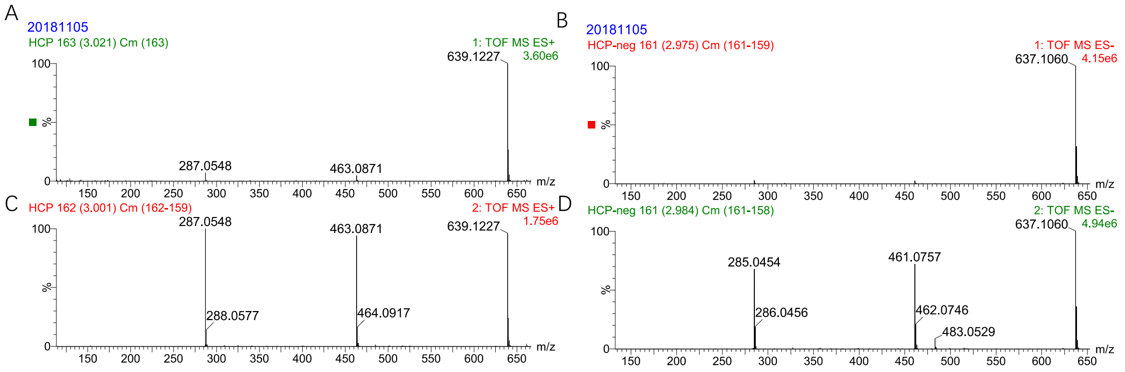

Figure S4. Mass spectrogram of luteolin-3′,7-O-diglucuronides at 3.00 min (**A**) positive ionization mode at low energy channels; (**B**) negative ionization mode at low energy channels; (**C**) positive ionization mode at high energy channels; (**D**) negative ionization mode at high energy channels.

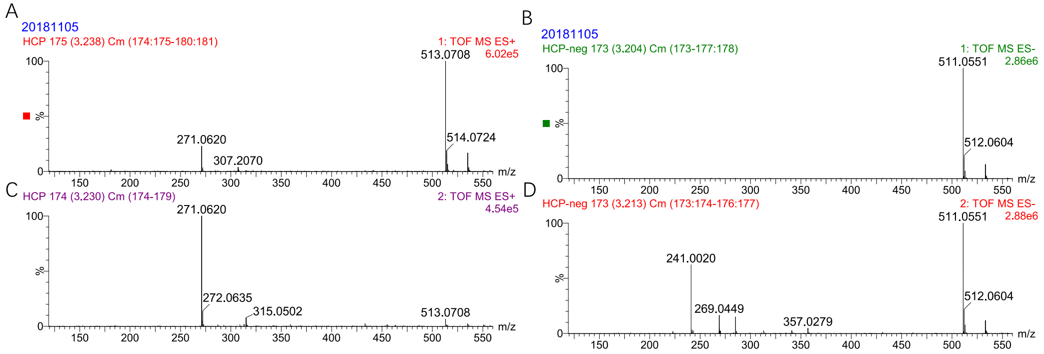

Figure S5. Mass spectrogram of diphlorethohydroxycarmalol at 3.22 min (**A**) positive ionization mode at low energy channels; (**B**) negative ionization mode at low energy channels; (**C**) positive ionization mode at high energy channels; (**D**) negative ionization mode at high energy channels.

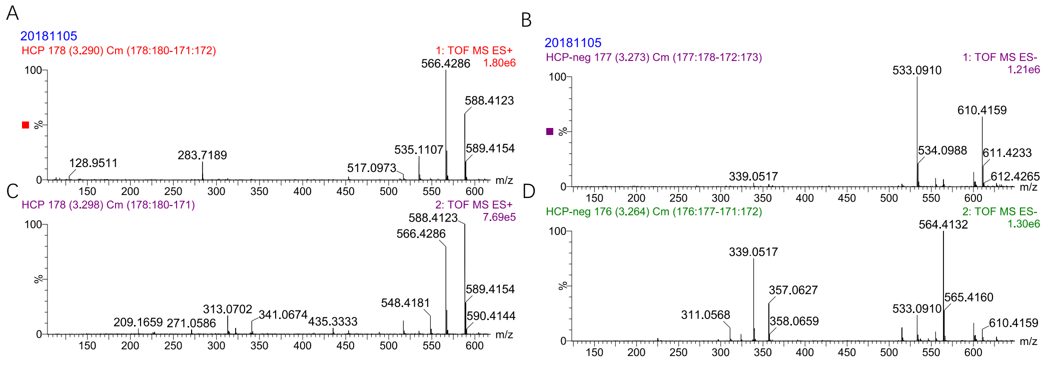

Figure S6. Mass spectrogram of cyclo (L-leucyl-L-leucyl-L-leucyl-L-leucyl-L-leucyl) at 3.27 min (**A**) positive ionization mode at low energy channels; (**B**) negative ionization mode at low energy channels; (**C**) positive ionization mode at high energy channels; (**D**) negative ionization mode at high energy channels.

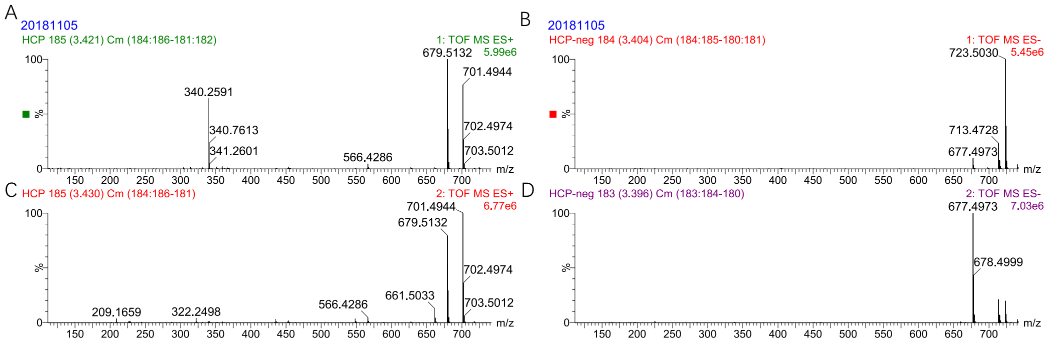

Figure S7. Mass spectrogram of cyclo (L-leucyl-L-leucyl-L-leucyl-L-leucyl-L-leucyl-L-leucyl) at 3.42 min (**A**) positive ionization mode at low energy channels; (**B**) negative ionization mode at low energy channels; (**C**) positive ionization mode at high energy channels; (**D**) negative ionization mode at high energy channels.

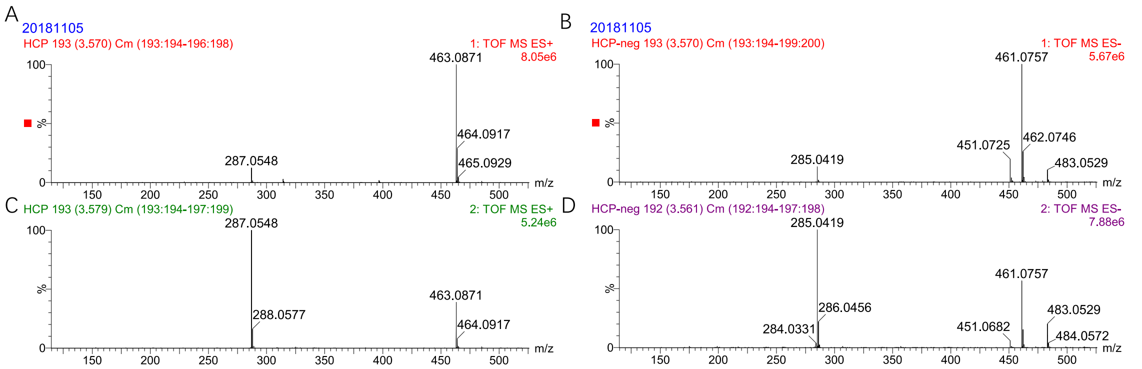

Figure S8. Mass spectrogram of luteolin-7-O-glucuronide at 3.55 min (**A**) positive ionization mode at low energy channels; (**B**) negative ionization mode at low energy channels; (**C**) positive ionization mode at high energy channels; (**D**) negative ionization mode at high energy channels.

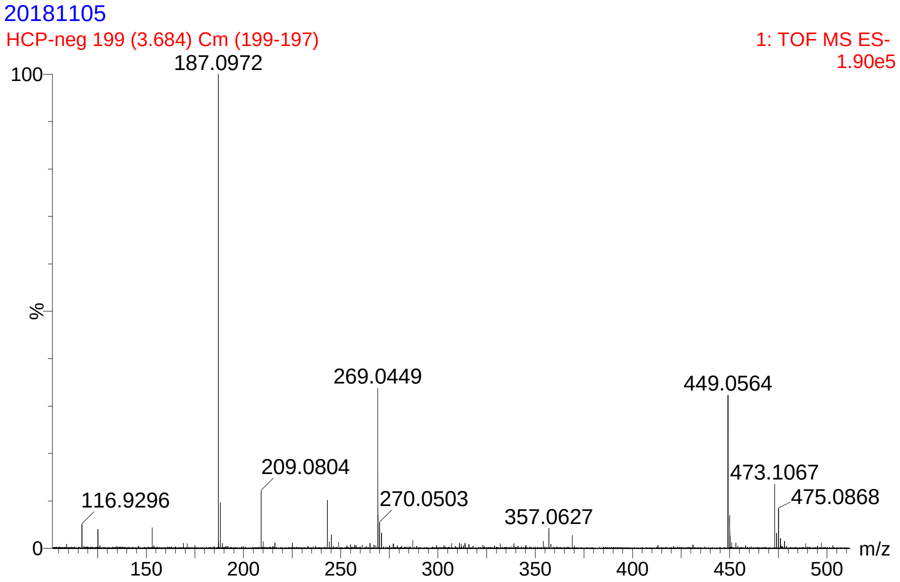

Figure S9. Mass spectrogram of chrysoeriol-7-O-glucuronide at 3.66 min negative ionization mode at low energy channels.

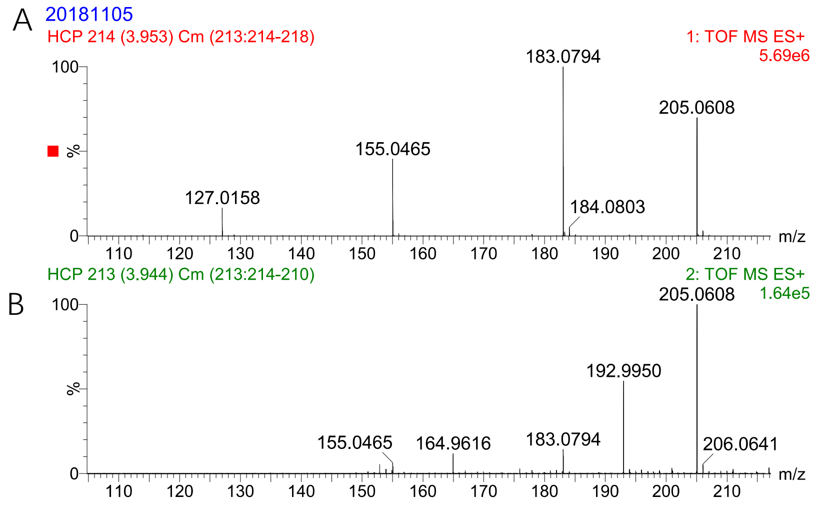

Figure S10. Mass spectrogram of 6-hydroxy-1,3-divinyldihydropyrimidine-2,4,5(3H)-trione at 3.95min (**A**) positive ionization mode at low energy channels; (**B**) postive ionization mode at high energy channels.

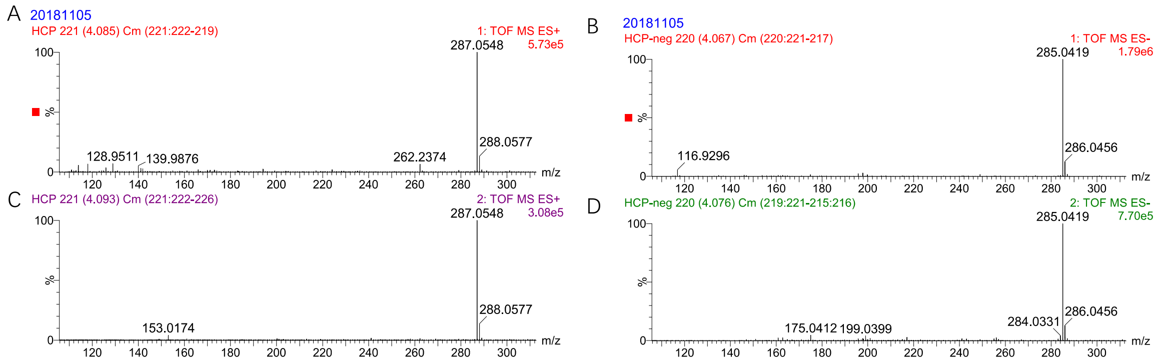

Figure S11. Mass spectrogram of luteolin at 4.07min (**A**) positive ionization mode at low energy channels; (**B**) negative ionization mode at low energy channels; (**C**) positive ionization mode at high energy channels; (**D**) negative ionization mode at high energy channels.

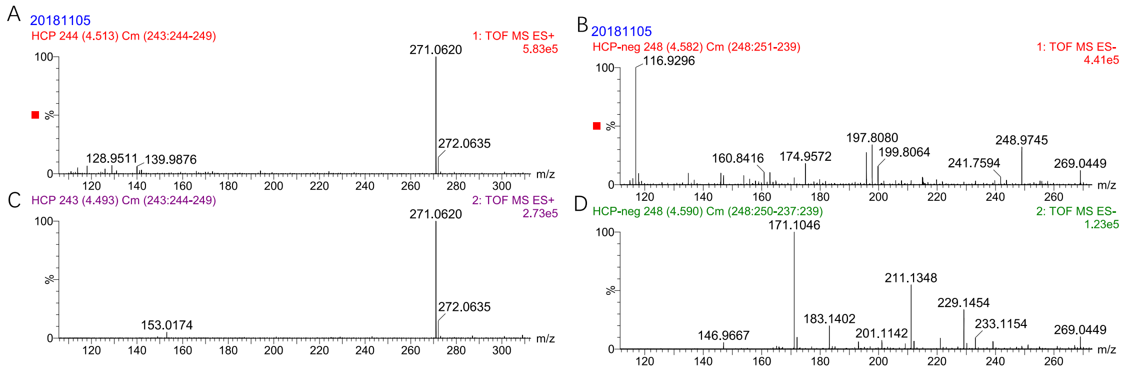

Figure S12. Mass spectrogram of apigenin at 4.48min (**A**) positive ionization mode at low energy channels; (**B**) negative ionization mode at low energy channels; (**C**) positive ionization mode at high energy channels; (**D**) negative ionization mode at high energy channels.

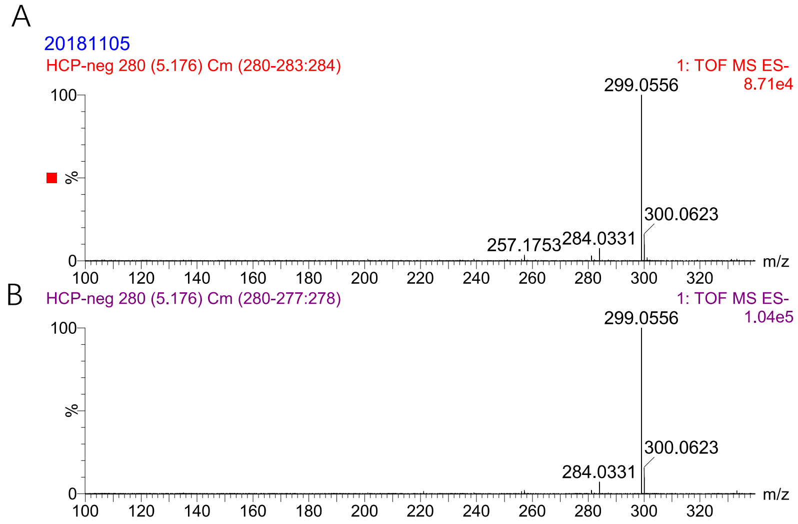

Figure S13. Mass spectrogram of chrysoeriol at 5.18 min (**A**) negative ionization mode at low energy channels; (**B**) negative ionization mode at high energy channels.

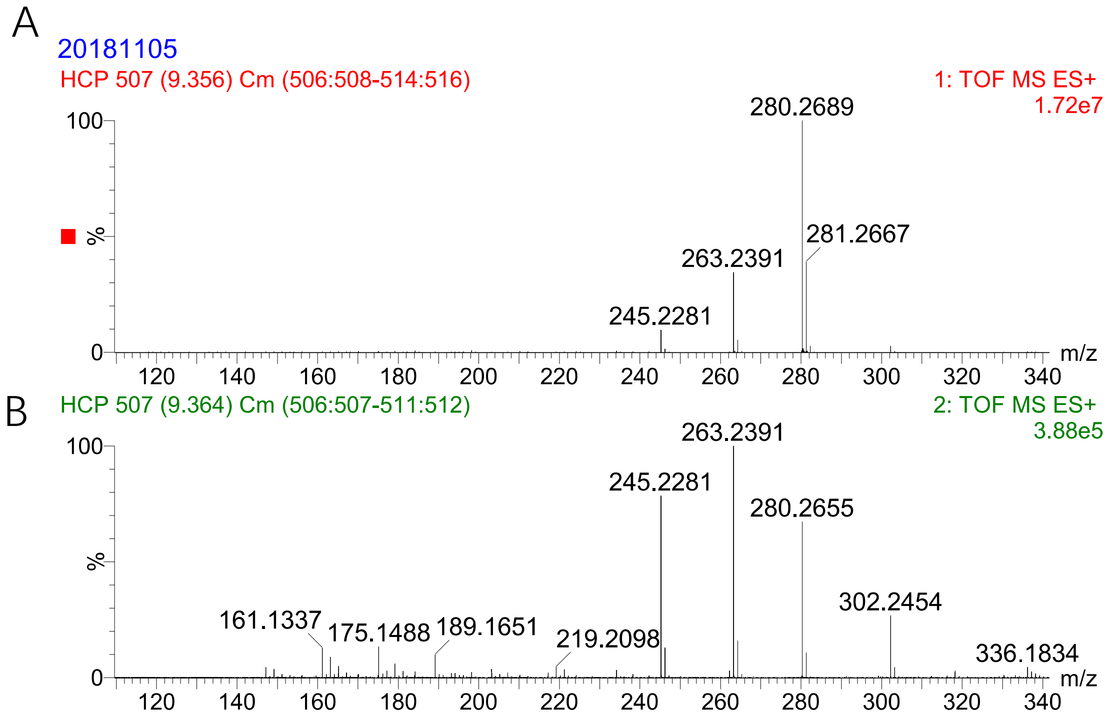

Figure S14. Mass spectrogram of (9E,12Z)-octadeca-9,12-dienamide at 9.36 min (**A**) positive ionization mode at low energy channels; (**B**) positive ionization mode at low energy channels.

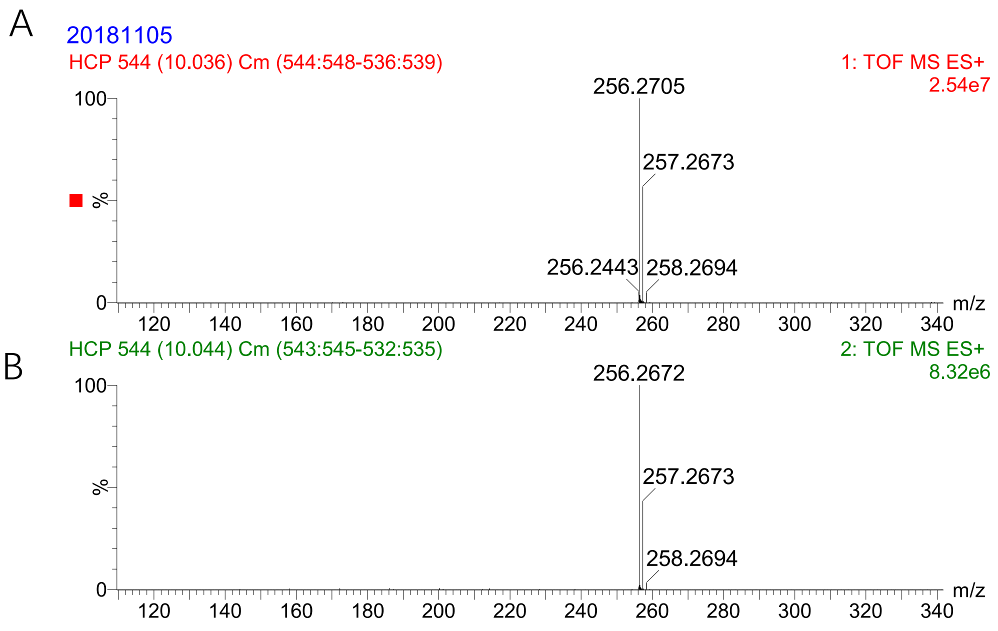

Figure S15. Mass spectrogram of palmitamide at 10.04 min (**A**) positive ionization mode at low energy channels; (**B**) positive ionization mode at low energy channels.
